# Candidemia in Southern Poland (2017–2022): Multicenter Analysis of Species Distribution and Antifungal Susceptibility

**DOI:** 10.3390/jof12030212

**Published:** 2026-03-15

**Authors:** Magdalena Namysł, Magdalena Skóra, Monika Pomorska-Wesołowska, Małgorzata Romanik, Wioletta Świątek-Kwapniewska, Piotr Serwacki, Iwona Pawłowska, Aldona Olechowska-Jarząb, Jadwiga Wójkowska-Mach

**Affiliations:** 1 Department of Microbiology, University Hospital in Krakow, Jakubowskiego 2 Street, 30-688 Krakow, Poland; mnamysl@su.krakow.pl (M.N.); aldona.olechowska-jarzab@uj.edu.pl (A.O.-J.); 2 Department of Microbiology, Faculty of Medicine, Jagiellonian University Medical College, Czysta 18 Street, 31-121 Krakow, Poland; magdalena.skora@uj.edu.pl; 3 Department of Microbiology, KORLAB Medical Laboratories in Ruda Ślaska, 41-712 Ruda Śląska, Poland; monikapw@op.pl; 4 Department of Medical Microbiology, Faculty of Medical Sciences in Katowice, Medical University of Silesia, Medykow 18, 40-752 Katowice, Poland; mromanik@sum.edu.pl; 5 Faculty of Medicine and Health Sciences, University of Tarnów, 33-100 Tarnów, Poland; wkwapniewska@lukasz.med.pl; 6 St. Luke’s Provincial Hospital, 33-100 Tarnów, Poland; pserwacki@lukasz.med.pl; 7 Division of Microbiology, St. Barbara Specialized Regional Hospital No. 5 in Sosnowiec, 41-214 Sosnowiec, Poland; ipawlowska@wss5.pl

**Keywords:** candidemia, *Candida* spp., invasive candidiasis, antifungal susceptibility, fluconazole

## Abstract

*Candida* fungi are among the most common human fungal pathogens and invasive candidiasis is one of the predominant invasive mycoses that mainly affects hospitalized patients with suppression of the immunological system and breaches in skin or mucosal barriers. Rapid diagnosis and implementation of appropriate antifungal treatment are key to achieving recovery. In recent years, attention has been drawn to the increasing significance of *Candida* species other than *C. albicans*, in particular *C. auris* and fluconazole-resistant *C. parapsilosis*. The aim of this work was to present the species spectrum and drug susceptibility of 570 *Candida* strains isolated from candidemia cases diagnosed in patients hospitalized in southern Poland in the period 2017–2022. The results of *Candida*-positive blood cultures obtained from five hospitals were analyzed. *C. albicans* was the most common species, accounting for 42.6% of all strains, followed by *Nakaseomyces glabratus* (formerly *C. glabrata*) and *C. parapsilosis* complex—22.1% and 18.8%, respectively. No *C. auris* was found. Fluconazole resistance was found in 4.9% of *C. albicans* strains, 34.7% of *N. glabratus* strains, and 8.7% of *C. parapsilosis* complex strains.

## 1. Introduction

*Candida* fungi are among the most common human fungal pathogens responsible for a wide spectrum of infections, ranging from superficial skin and mucous membranes mycoses to severe deep mycoses affecting various organs and often associated with hematogenous dissemination with concomitant candidemia [1,2]. Most infections are of endogenous origin, but exogenous infections also occur, including cases of horizontal transmission of pathogens in the hospital environment. The latter concern in particular *Candida auris* [3,4], but has also been reported for other *Candida* species [5,6,7].

Invasive candidiasis, which is one of the predominant invasive fungal infections [8], occurs especially in critically ill patients, who present significant factors contributing to invasive fungal diseases, associated with suppression of the immunological system and/or breaches in skin or mucosal barriers which enable microbiota translocation [2,9]. Most cases are hospital-acquired infections, which are more or less directly caused by the medical procedures used during the patient’s hospital stay. It is estimated that *Candida* may constitute 5 to 15% of the pathogens responsible for hospital-acquired bloodstream infection in intensive care unit (ICU) patients [10]. Community-acquired candidemia accounts for less than a quarter [2].

The genus *Candida* includes heterogeneous species of different phylogeny. This results in different virulence of these species, which is reflected in i.a. pathogenicity, horizontal transmission capabilities, and also different sensitivity to antifungal drugs, both in the context of natural resistances and the ability to acquire resistance after exposure to antimycotic substances. Continuously, the dominant species worldwide in candidaemia is *Candida albicans*, but for many years, an increase in the prevalence of other *Candida* species has also been observed, among which the following dominate: *Candida glabrata* (currently *Nakaseomyces glabratus*), *Candida parapsilosis* species complex, *Candida krusei* (currently *Pichia kudriavzevii*), *Candida tropicalis*, and *C. auris* [11]. The epidemiology of invasive candidiasis and candidemia varies in different geographical areas and within various patient populations, depending on age, primary diseases, and other risk factors, as well as patients’ previous contact with antimycotic medication [2,11,12,13].

Due to the previously mentioned interspecies differences, infections caused by individual representatives of the *Candida* genus may be associated with various therapeutic problems and the knowledge of the species causing the infection allows prediction of the effectiveness of empirical therapy and probable outcome. However, the choice of the appropriate drug is complicated by the growing problem of acquired drug resistance of fungi as a consequence of therapeutic errors and because of the exposure of fungi to non-medical sources of antimycotic substances, i.e., plant protection products used in agriculture [14,15]. Currently, three groups of antifungal drugs are used in the treatment of invasive candidiasis: polyenes (amphotericin B), azoles and echinocandins, with the latter recommended as first-line drugs [2,16]. The greatest challenge in therapy is azole resistance, most frequently noted in *Candida* strains. Resistance to amphotericin B and echinocandins is rather rare or applies to selected species, e.g., *Candida lusitaniae* for amphotericin B, and *C. parapsilosis* for echinocandins. In recent years, special attention has been paid to infections caused by multidrug-resistant *C. auris* species. This species spreads globally and is reported in various regions of the world as a cause of difficult-to-control and to cure epidemic infections in hospital units, which have become even more intense during the COVID-19 pandemic [17,18,19]. A disturbing phenomenon in some regions is also the epidemic occurrence of *C. parapsilosis* strains resistant to fluconazole [5,20]. *C. parapsilosis* strains are usually characterized by elevated minimum inhibitory concentration (MIC) values for echinocandins, which may favor the emergence of resistance to this antifungal group, and additional resistance to fluconazole causes a dramatic reduction in the number of drugs that can be used to treat infections.

Poland, which is a country of about 40 million people, located in central Europe, with good access to medical services, is a blank spot on the world map of fungal species causing infections and their drug susceptibility profiles. Due to the relatively close location of countries where epidemics caused by *C. auris* and fluconazole-resistant *C. parapsilosis* strains are reported (i.e., Italy [21] or Spain [22,23]), it seemed desirable to analyze the etiological factors of candidemia in the period preceding and including the COVID-19 pandemic, which favored the spread of serious fungal infections. The objective of this study is to characterize the species spectrum of *Candida* isolates recovered from cases of candidemia in Polish hospitals and to evaluate their susceptibility to commonly used antifungal drugs. This investigation aims to identify the predominant species involved in bloodstream infections and to monitor potential shifts in antifungal resistance patterns, providing insights relevant to clinical management and therapeutic decision-making.

## 2. Materials and Methods

### 2.1. Study Area and Time Frame

This study involved a retrospective analysis of candidemia cases based on blood culture results obtained from patients presenting with clinical signs of sepsis or septic shock between 1 January 2017 and 31 December 2022, in five hospitals located in southern Poland (Hospitals A–E):

- Tertiary academic center, a large multispecialty university hospital providing comprehensive adult care (~1300 inpatient beds, including 60 ICU beds);
- Large provincial general hospital, a multispecialty facility with ~700 inpatient beds, including 32 ICU beds;
- Regional high-specialist trauma center, a teaching multiprofile hospital with designated trauma services (~600 inpatient beds, including 18 ICU beds);
- Municipal multi-profile complex, adult and pediatric secondary/tertiary care, ~550 inpatient beds, including 8 adult ICU beds and 8 pediatric ICU beds;
- A single-profile geriatric, non-teaching hospital (~100 inpatient beds, without dedicated ICU beds).

Because a detailed case-by-case clinical verification was not feasible, predefined assumptions were applied:

1. Only candidemia episodes confirmed from peripherally collected venous or arterial blood samples were included in the analysis. Blood cultures drawn through central venous catheters (CVCs) were excluded, as isolates recovered exclusively from CVC-derived specimens are more likely to represent catheter colonization rather than true bloodstream infection [24].
2. All positive blood cultures with *Candida* spp. obtained within one year of the index candidemia episode were considered part of the same infectious episode. Isolation of *Candida* spp. occurring more than one year after the initial episode was classified as a new candidemia event. The one-year timeframe was adopted by the authors specifically for the purposes of this publication to minimize misclassification of prolonged or recurrent fungemia arising from a single pathogenic process as separate episodes, given the heterogeneity of disease courses and the potential for delayed bloodstream clearance or deep-seated involvement [25].

Data for the analysis, originating from cases with blood cultures positive for *Candida* species, were exported from laboratory information systems as aggregated statistical reports and included the following variables: patient age and sex, hospital ward, identified *Candida* species, and antifungal susceptibility results.

This multicenter study focused on species distribution and antifungal susceptibility profiles. Data from participating centers were pooled and analyzed to identify epidemiological patterns in the distribution of *Candida* species and their resistance profiles. Additionally, because the majority of candidemia cases originated from a single institution—Hospital A—and because laboratory procedures differed substantially among the centers, year-to-year comparisons of *Candida* species distribution and antifungal susceptibility patterns were based exclusively on data from Hospital A. This strategy ensured methodological uniformity and provided a more reliable assessment of temporal trends in species occurrence and antifungal resistance.

### 2.2. Culture, Identification, and Drug Susceptibility Testing Methods

The initial diagnostic workflow in all participating laboratories followed standard procedures for blood culture processing [26,27]. Immediately after venipuncture, blood samples were inoculated into liquid culture bottles dedicated to either the BacT/ALERT system (bioMérieux, Lyon, France) or the BD BACTEC™ system (Becton Dickinson, Franklin Lakes, NJ, USA) and incubated according to the manufacturers’ instructions. When a blood culture bottle signaled positive, the presence of yeasts was first confirmed by direct microscopy. Subsequently, standard subculturing was performed, including inoculation onto fungal isolation media such as Sabouraud Dextrose Agar supplemented with antibiotics. Subsequent diagnostic stages exhibited substantial heterogeneity depending on the healthcare facility and varied over the analyzed time (Table S1 Supplementary Information). Species-level identification of *Candida* isolates was performed using the automated systems based on biochemical properties of isolates—VITEK^®^ 2 YST (bioMérieux, Lyon, France) or BD Phoenix™ System M50 (Becton Dickinson, Franklin Lakes, NJ, USA), and/or mass spectrometry-based platforms—VITEK^®^ MS (bioMérieux, Lyon, France), and MALDI Biotyper (Bruker Daltonics, Bremen, Germany). Antifungal susceptibility testing was conducted using multiple commercial platforms, including: VITEK^®^ 2 AST-YS08 (bioMérieux, Lyon, France), Sensititre™ YeastOne™ YO10 (Thermo Fisher Scientific, Waltham, MA, USA), MICRONAUT-AM (MERLIN Diagnostika GmbH, Bornheim, Germany), ATB Fungus 3 INT (bioMérieux, Lyon, France), FUNGITEST^®^ (BIO-RAD, Marnes La Coquette, France), E-test (bioMérieux, France), and Liofilchem^®^ MIC Test Strip and RPMI agar (Liofilchem, Roseto degli Abruzzi, Italy) (Table S1 Supplementary Information). MICs were determined according to the manufacturers’ protocols. Regardless of the platform used, MIC values obtained for each antifungal agent–*Candida* isolate combination were interpreted according to the European Committee on Antimicrobial Susceptibility Testing (EUCAST) clinical breakpoints valid for the respective year, as provided in the EUCAST Breakpoint Tables for Interpretation of MICs for Antifungal Agents (Clinical breakpoints—fungi; versions 9.0, 8.1, and 7.0) [28]. This interpretation enabled classification of isolates into susceptible (S), intermediate (I), and resistant (R) categories. Throughout the manuscript, the term “susceptibility” refers to isolates with MIC values falling within the S or I categories. Due to incomplete susceptibility data across centers, the final analysis was restricted to four antifungal drugs: amphotericin B, fluconazole, voriconazole, and anidulafungin, and susceptibility results for all remaining antifungal drugs were not included in the analysis.

## 3. Results

### 3.1. Species Distribution

In total, 561 cases of candidemia were included in the analysis (301 women and 260 men). The age of female patients ranged from 20 to 94 years (mean: 66 years), while for male patients it ranged from 18 to 91 years (mean: 62 years). Nine patients exhibited co-infections involving two *Candida* species, resulting in a total of 570 isolates available for further examination. According to hospitalization ward data, 46.7% of cases originated from Intensive Care Units, 36.7% from Internal Medicine wards, and 16.6% from Surgical wards, indicating that the majority of episodes occurred in severely ill individuals or those with complex underlying medical conditions.

The most common species isolated was *Candida albicans* (*n* = 243, 42.6%), followed by *Nakaseomyces glabratus* (formerly *C. glabrata*) (*n* = 126, 22.1%) and the *C. parapsilosis* complex (*n* = 107, 18.8%) (Table 1).

The distribution of *Candida* species differed between the hospitals. The proportion of *C. albicans* reached the highest value in Hospital B (55%) and remained relatively high in Hospitals A, C, and D, where it accounted for 45.20%, 36.84%, and 42.20% of isolates, respectively. In contrast, Hospital E showed a distinctly lower prevalence of *C. albicans* at only 18.64%. In Hospitals A–D, the overall species profiles of candidemia were broadly similar, with *C. albicans* either dominating the species distribution. Non-*albicans* species represented approximately 45–53% of isolates in these units. However, Hospital E demonstrated a pronounced shift, with non-*albicans* species comprising 77.96% of all isolates, with high prevalence of the *C. parapsilosis* complex (38.98%) and *C. tropicalis* (10.12%). The distribution of other non-*albicans* species—such as *N. glabratus*, *C. lusitaniae*, and *C. krusei*—showed smaller fluctuations between studied hospitals (Table 1).

### 3.2. Temporal Trends in Candida Species Distribution

**Figure 1:**
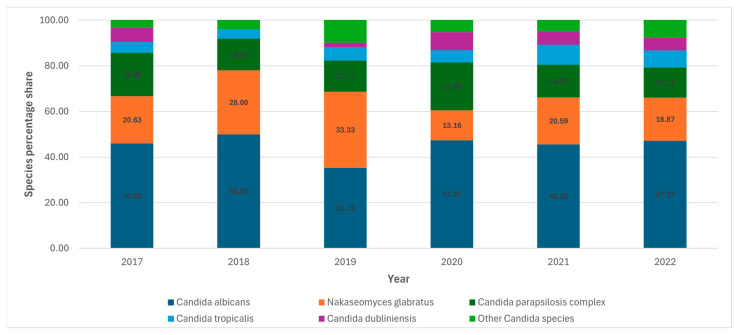
Temporal trends in the species distribution of *Candida* isolates from candidemia in Hospital A (2017–2022).

Temporal trends in the distribution of *Candida* species were assessed using data from Hospital A, which reported the largest number of candidemia episodes during the study period. An examination of cases recorded between 2017 and 2022 demonstrated only modest year-to-year variation in the species distribution, without evidence of a consistent directional trend (Figure 1; Supplementary Information Table S2).

Throughout the six-year observation period, *C. albicans* consistently represented the predominant etiologic agent of candidemia. Its relative contribution remained remarkably stable at approximately 50%, indicating a persistent dominance of this species in the local epidemiological landscape. Only a single deviation from this pattern was observed in 2019, when the proportion of *C. albicans* temporarily declined. This isolated fluctuation does not appear to reflect a sustained epidemiological shift but rather a transient disturbance within an otherwise stable long-term distribution pattern.

Among non-*albicans* species, *N. glabratus* represented the second most prevalent species throughout the study period, though its share varied considerably. Its proportion rose from 20.6% of isolates in 2017 to 28.0% in 2018 and peaked at 33.3% in 2019. A marked decline followed in 2020 (13.2%), after which the species regained a stable mid-range frequency of ~20% in 2021–2022.

The *C. parapsilosis* exhibited moderate but fluctuating prevalence. Its proportion remained within approximately 12–16% in most years but increased noticeably in 2017 and 2020, reaching about 19% and 21% of all candidemia isolates, respectively.

Among the less common species, several appeared only sporadically and did not demonstrate any consistent temporal patterns. *C. dubliniensis* constituted 4–6% of isolates in selected years. *C. tropicalis* accounted for 3–6% of cases with no clear trend, though its highest proportions appeared in the last two years (2021–2022). Other *Candida* species occurred only sporadically and at very low frequencies (0–2%), without contributing significantly to the temporal dynamics.

### 3.3. Antifungal Susceptibility Profiles

When considering all isolates collected across the participating hospitals, the distribution of susceptible isolates varied across *Candida* species and antifungal drugs (Table 2). Among *C. albicans*, nearly all isolates were susceptible to amphotericin B and anidulafungin (98.2% and 99.5%), while slightly lower proportions were observed for fluconazole and voriconazole (95.1% and 94.6%). In *Nakaseomyces glabratus*, amphotericin B and anidulafungin showed consistently high percentages of susceptible isolates (98% and 100%), whereas fluconazole susceptibility was substantially lower, with only 65.3% of isolates classified as susceptible. For *Pichia kudriavzevii*, 75% of isolates were susceptible to amphotericin B, while no isolates were susceptible to fluconazole, consistent with intrinsic resistance; all tested isolates were susceptible to anidulafungin. The *C. parapsilosis* complex displayed complete susceptibility to amphotericin B, with lower percentages of isolates susceptible to fluconazole, voriconazole, and anidulafungin (91.3%, 90.4%, and 93.1%, respectively). In *C. tropicalis*, all isolates were susceptible to amphotericin B, while the proportions of isolates susceptible to fluconazole and voriconazole were reduced (80% and 75%); all isolates tested for anidulafungin were susceptible (Table 2).

**Figure 2:**
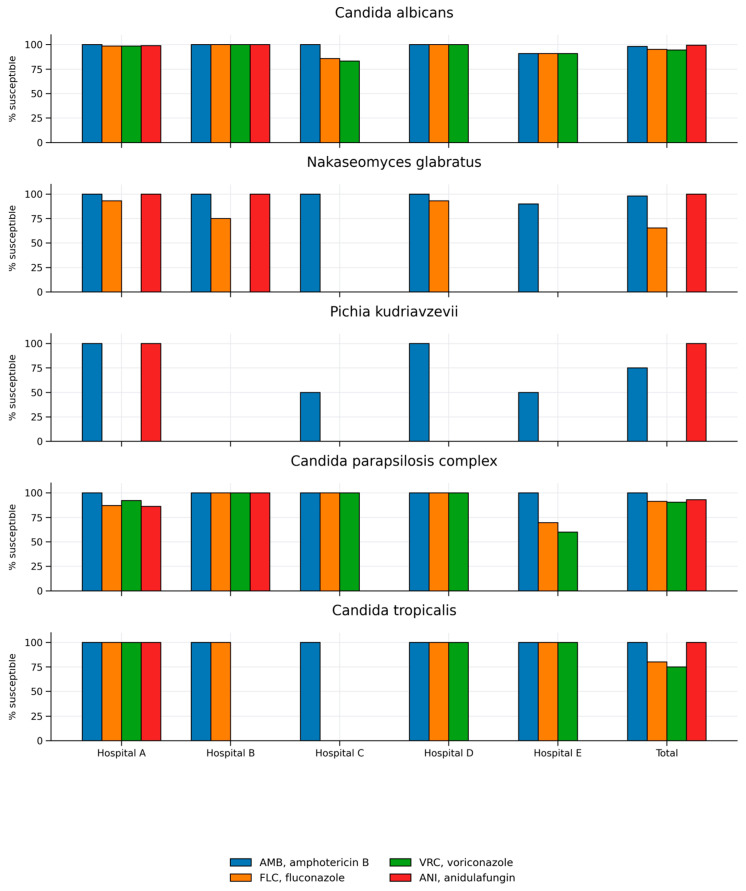
Distribution of susceptible (S and I susceptibility category) *Candida* isolates to four antifungal drugs across hospitals A–E. AMB, amphotericin B; FLC, fluconazole; VRC, voriconazole; ANI, anidulafungin.

Inter-hospital analysis revealed notable variability in the rate of susceptible isolates among major *Candida* species. *C. albicans* showed uniformly high rates of amphotericin B– and anidulafungin-susceptible isolates, with reduced azole susceptibility observed in Hospitals C and E. *N. glabratus* demonstrated substantial variability in fluconazole susceptibility, ranging from a high rate of susceptible isolates (Hospitals A and D) to a moderate rate (Hospital B) and absence of susceptible isolates (Hospital E), while maintaining susceptibility to amphotericin B. In *P. kudriavzevii*, amphotericin B susceptibility rates varied considerably, dropping to 50% in Hospitals C and E, alongside the expected universal fluconazole resistance. The *C. parapsilosis* complex exhibited wide inter-hospital differences in azole susceptibility rates, with full susceptibility in Hospitals B–D and lower values in Hospitals A and E. *C. tropicalis* remained uniformly susceptible to amphotericin B, with azole-nonsusceptibility observed only in the single isolate from Hospital C (Table 2, Figure 2).

### 3.4. Temporal Trends in Antifungal Susceptibility Profiles

A detailed MIC analysis was performed for four antifungal agents—fluconazole, voriconazole, anidulafungin, and amphotericin B—in relation to the three most common species causing candidemia in our cohort: *C. albicans*, *N. glabratus*, and *C. parapsilosis*, isolated in Hospital A. During the six-year observation period, changes in the methods used for MIC determination were observed, which affected the comparability of results over time. As the applied methods differed in their MIC ranges, methodological variability likely contributed to the observed variation in MIC values. This effect was particularly notable for anidulafungin, for which two different MIC determination methods were used concurrently during part of the study period. Consequently, MIC values presented in Table 3 should be interpreted in light of this methodological heterogeneity.

### 3.5. Candida albicans

The MIC distributions for *C. albicans* isolates remained largely stable throughout the six-year period, with consistently low values across all tested antifungal agents. For fluconazole, most *C. albicans* isolates exhibited MIC 0.5 mg/L, forming a steady mode that was reproducible across consecutive years. An exception is the year 2017, in which strains with an MIC value of 1.0 mg/L predominated. Higher MIC values were occasionally recorded and did not form a temporal trend, indicating no apparent development of reduced susceptibility within the population. The overall distribution is consistent with the well-recognized susceptibility of *C. albicans* to fluconazole and suggests preserved activity of this agent in the study setting.

Voriconazole MICs were similarly concentrated at low levels, with most isolates falling between 0.06 and 0.125 mg/L in all study years. Importantly, no increase in higher MIC categories was observed, reinforcing the consistent susceptibility of *C. albicans* to voriconazole.

The anidulafungin MIC distribution revealed uniformly low values, with nearly all isolates showing MICs between 0.002 and 0.016 mg/L. A noticeable increase in the number of *C. albicans* isolates exhibiting higher MIC values to anidulafungin was observed in the last two years of the study. Although these elevated MICs remained infrequent, their emergence may suggest early variability in echinocandin susceptibility within the population.

**Figure 3:**
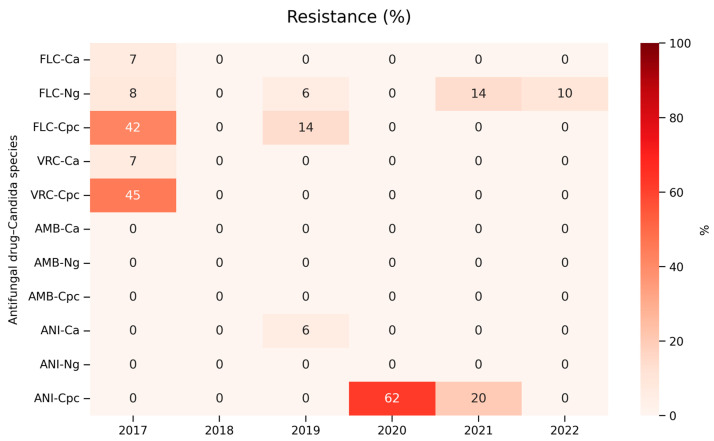
The percentage of resistant *Candida* isolates for each antifungal drug–species combination from 2017 to 2022 based on Hospital A data. Ca—*Candida albicans*, Ng—*Nakaseomyces glabratus*, Cpc—*Candida parapsilosis* complex, AMB, amphotericin B; FLC, fluconazole; VRC, voriconazole; ANI, anidulafungin.

Amphotericin B demonstrated a similarly stable pattern, with a prominent peak at 0.25–0.5 mg/L (Table 3, Figure 3).

### 3.6. Nakaseomyces glabratus

The MIC profiles of *N. glabratus* differ significantly from those of *C. albicans*, reflecting its intrinsic reduced susceptibility to azoles and its known capacity to develop echinocandin resistance. The distribution of fluconazole MICs for most strains is in the range of up to 16 mg/L, with some isolates reaching 32–256 mg/L.

Voriconazole MICs demonstrated wide variability; however, the majority of isolates displayed MIC values within the 0.125–0.25 mg/L range.

Anidulafungin MICs were predominantly low during the earlier years of the study (typically 0.004–0.016 mg/L), but an increase in the number of isolates with higher MICs was noted in the final two years, including values of 0.03–0.06 mg/L and a single isolate reaching 0.125 mg/L.

Amphotericin B MICs remained comparatively stable across the entire study period, clustering mainly around 0.25–0.5 mg/L, with a single strain of MIC value at 1 mg/L (Table 3, Figure 3).

### 3.7. Candida parapsilosis

Among *C. parapsilosis* isolates, fluconazole MIC values were predominantly clustered around 0.5–1 mg/L, with occasional isolates reaching 2–8 mg/L, and a single high outlier at 256 mg/L observed in 2017. No trend indicating the emergence of resistance over time was observed.

Voriconazole MICs showed a narrower distribution, with most isolates exhibiting values between 0.094 and 0.125 mg/L, while higher MICs (0.25–0.75 mg/L) occurred rarely and sporadically across the study years. The consistently low MIC values observed across all study years suggest a retained wild-type susceptibility pattern.

Anidulafungin MIC values demonstrated a broad range characteristic of this species, with isolates distributed across 0.125–2 mg/L, and sporadic occurrences of higher MICs up to 8–12 mg/L, without a clear temporal trend.

Amphotericin B MICs remained relatively stable, with most isolates presenting values between 0.125 and 0.25 mg/L, accompanied by occasional higher values (0.5–1 mg/L), but without evidence of a sustained shift over time (Table 3, Figure 3).

## 4. Discussion

The present study provides a comprehensive overview of the species distribution of *Candida* isolates causing candidemia and their antifungal susceptibility profiles over a six-year period in patients hospitalized in five Polish hospitals. The selected study period (2017–2022) was deliberately chosen because it encompasses both the pre-pandemic years and the COVID-19 pandemic era. This interval is epidemiologically meaningful: during the pandemic, a substantial global increase in invasive fungal infections was observed, particularly involving *Candida* spp., *Aspergillus* spp., and *Mucorales*. In the specific context of candidemia, several studies documented changes in its epidemiology during the COVID-19 period, including shifts toward non-*albicans* species and altered antifungal susceptibility patterns. As in many recent surveillance studies, the traditional dominance of *C. albicans* is increasingly challenged by non-*albicans Candida* species, which collectively accounted for a substantial proportion of bloodstream infections in our cohort (non-*C. albicans* species accounted for more than half of candidemia species). This shift mirrors global trends showing a steady increase in non-*albicans* candidemia, notably *N. glabratus* (formerly *C. glabrata*) and the *C. parapsilosis* complex. Such a change carries important clinical implications, as several non-*albicans* species are associated with reduced susceptibility or emerging resistance to commonly used antifungal agents [29,30,31,32,33].

A notable feature of the local dataset is the fluctuation in the proportion of *N. glabratus*, which shows moderate variation between years (with peaks in 2018 and 2019 and declines thereafter). This is consistent with international data, where *N. glabratus* has been described as increasingly prevalent—particularly in older and heavily treated patient populations—while its relative frequency varies geographically. Literature indicates that this species often expands in settings with high azole exposure, and its rise has been documented in North America and parts of Europe, though its incidence remains lower in Mediterranean and Latin American cohorts [34,35,36].

In our analysis, changes in the proportion of *C. parapsilosis* over time could be observed. The species shows a transient increase in 2020 (when the highest number of SARS-CoV-2-related infections were recorded), followed by a marked reduction in 2021 and a partial rebound thereafter (Figure 1). In 2020, the percentage of infections caused by *C. parapsilosis* was higher than that caused by *N. glabratus* (21.05% vs. 13.16%). In the remaining years, the proportions are reversed. These fluctuations mirror findings from recent long-term epidemiological analyses showing that *C. parapsilosis* incidence is highly variable and strongly influenced by local factors such as catheter use, parenteral nutrition, caspofungin usage, and potential clonal transmission in healthcare settings. Several global studies have noted increasing *C. parapsilosis* rates in particular regions—including Southern Europe and Latin America—while others report stability or decline, emphasizing the role of local epidemiology and infection control practices [37,38,39]. The distribution of *Candida* species also varied substantially between hospitals, reflecting localized epidemiological pressures, patient characteristics, and patterns of care. Our findings emphasize that candidemia ecology is not uniform across the hospital and that targeted infection control interventions may be necessary in units exhibiting distinct species patterns.

Importantly, the study period (2017–2022) encompasses the COVID-19 pandemic, which had profound and well-documented effects on fungal epidemiology worldwide [19,40,41]. During the pandemic years—particularly 2020 and 2021—many centers reported increased candidemia incidence and altered species distribution driven by prolonged ICU stays, extensive use of broad-spectrum antibiotics, corticosteroids, immunomodulatory therapies, and high device burden (mechanical ventilation, central venous catheters) [29,42,43,44]. These factors created conditions conducive to invasive candidiasis and may partially explain the temporal shifts observed in our dataset. Moreover, disruptions to staffing, infection control practices, and patient flow during the pandemic could have contributed to local fluctuations in non-*albicans* species. Reports support that the pandemic has driven increased incidence and a higher proportion of non-*albicans Candida* fungemia [45]. Although our data do not show a dramatic surge in species associated with COVID-19 ICUs, such as *C. tropicalis* or *N. glabratus*, the year-to-year variability aligns with global observations describing pandemic-related perturbations in candidemia ecology.

Minor species such as *C. tropicalis*, *C. kefyr*, *C. orthopsilosis*, and *Pichia kudriavzevii* appear only sporadically in the dataset, which matches global observations that uncommon and emerging yeasts represent a small but growing proportion of invasive infections. Recent reviews highlight a rising number of reports involving uncommon species, partly due to improved diagnostic methods such as MALDI-TOF and routine molecular identification. However, their contribution to the overall candidemia burden remains modest in most centers [46].

The European Union/European Economic Area (EU/EEA) countries face rapid dissemination of *C. auris* [47]. The latest European Centre for Disease Prevention and Control (ECDC) review documented a sharp increase in *C. auris* isolation since 2020 (in 2023, 18 EU/EEA countries reported a total of 1346 cases), with local endemicity in some countries, and highlighted gaps in laboratory preparedness and surveillance [48]. The burden was highest in Spain, Greece, Italy, Romania, and Germany. Although *C. auris* was not detected in our cohort, the European experience underscores the need for diagnostic vigilance (validated MALDI-TOF, PCR/sequencing), readiness for outbreak investigations, and strict infection control procedures to prevent transmission. Notably, Poland currently lacks unified, nationwide standards governing the diagnosis, surveillance, and control of invasive fungal infections, including *C. auris*. This absence of standardized national guidelines poses a tangible public health risk and underscores the urgent need to implement coordinated diagnostic protocols and surveillance frameworks to ensure early detection and effective containment of emerging fungal threats. In line with these concerns, our analysis highlights substantial heterogeneity in the diagnostic methods used across laboratories for fungal identification and antifungal susceptibility testing, further emphasizing the need for harmonized national standards.

Antifungal susceptibility testing in our study confirmed expected patterns: high activity of amphotericin B and anidulafungin against *C. albicans* and *N. glabratus*, reduced fluconazole efficacy against *N. glabratus*, and intrinsic resistance of *C. krusei* to fluconazole. These findings are consistent with the Infectious Diseases Society of America (IDSA) and EUCAST guidelines [32,49]. Interpretation of MIC values requires species-specific consideration (e.g., higher echinocandin MICs for *C. parapsilosis*) and awareness of ongoing EUCAST/CLSI breakpoint updates. At the species level, the *C. parapsilosis* complex deserves particular attention. A recent meta-analysis of 71 studies demonstrated a dramatic rise in fluconazole resistance, from ~11.6% before 2016 to ~36.7% in 2016–2022, with considerable regional heterogeneity [50]. The European Confederation of Medical Mycology (ECMM) surveillance (2018–2022) confirmed clusters of fluconazole-resistant strains and sporadic *FSK* mutations associated with reduced echinocandin susceptibility [31]. In our cohort, fluconazole susceptibility within the *C. parapsilosis* complex remained high, which differentiates Poland from many other regions; nevertheless, vigilant monitoring of MIC values is required.

The most recent Centers for Disease Control and Prevention (CDC) data provides one of the most detailed national epidemiological assessments of candidemia [51]. The report outlines the distribution of major species—including *C. albicans*, *N. glabratus*, *C. parapsilosis*, and *C. tropicalis*—and highlights ongoing challenges such as persistently high mortality and rising rates of azole and echinocandin resistance. Notably, the U.S. model illustrates the strengths of a coordinated national surveillance system: standardized diagnostics, mandatory reporting, and real-time trend monitoring facilitate early outbreak detection and ensure that shifts in epidemiology rapidly inform therapeutic guidelines and infection control strategies. Against this benchmark, the Polish situation—fragmented datasets, lack of a central registry, and methodological heterogeneity—illustrates the urgent need to establish a national candidemia surveillance program, integrated with European and global networks. Only such an approach will enable reliable trend analysis, rapid outbreak identification, and effective clinical response.

The strength of our study lies in its multicenter design and six-year horizon, providing a cross-sectional snapshot of candidemia epidemiology in a region with scarcely published data. Poland remains a “blind spot” in the global surveillance map: an earlier multicenter study (2013) covering 20 hospitals and 302 episodes already reported increasing non-*albicans* prevalence and ICU predominance [52]. Our results thus provide a valuable contemporary reference and reinforce the necessity of continuous, standardized surveillance.

## 5. Limitations

The analysis was performed using data extracted from laboratory information systems. Laboratory results were not verified against the patient’s clinical presentation and therefore do not represent a confirmed clinical diagnosis. Due to the retrospective nature of the study and the absence of routine strain archiving, repeat species identification and antifungal susceptibility testing could not be conducted to standardize laboratory methodology. This reliance on unvalidated laboratory data constitutes a methodological limitation that may influence the interpretation and generalizability of the findings.

## 6. Conclusions

Our study documents the current epidemiological landscape of candidemia in southern Poland—characterized by predominance of non-*albicans Candida*, preserved susceptibility to echinocandins and amphotericin B, and absence of *C. auris* during the study period. Collectively, these observations indicate that the species distribution and antifungal resistance patterns identified in Poland are broadly consistent with global epidemiological trends, notwithstanding the limited availability of national surveillance data. Importantly, no concerning developments were detected, including the emergence of *C. auris* or the appearance of fluconazole-resistant *C. parapsilosis*, both of which have been increasingly reported in other geographic regions. The major challenge in Poland lies in methodological heterogeneity and the absence of a unified surveillance system. Poland should pursue the development of a national candidemia registry integrated into European and global networks.

## Figures and Tables

**Table 1:** *Candida* species identified from candidemia cases based on data from five hospitals in Poland in a six-year period.

*Candida* Species	Hospital A	Hospital B	Hospital C *	Hospital D	Hospital E	Total
*Candida albicans* complex	161 (49.8%)	33 (55.0%)	9 (47.4%)	52 (47.7%)	13 (22.0%)	268 (47.0%)
*Candida albicans*	146 (45.2%)	33 (55%)	7 (36.8%)	46 (42.2%)	11 (18.6%)	243 (42.6%)
*Candida dubliniensis*	15 (4.6%)	0 (0.0%)	2 (10.5%)	6 (5.5%)	2 (3.4%)	25 (4.4%)
*Candida* other than the *C. albicans* complex	162 (50.2%)	27 (45.0%)	10 (52.6%)	57 (52.3%)	46 (77.9%)	302 (52.9%)
*Nakaseomyces glabratus* (formerly *Candida glabrata*)	73 (22.6%)	12 (20.0%)	2 (10.5%)	29 (26.6%)	10 (16.9%)	126 (22.1%)
*Pichia kudriavzevii* (formerly *Candida krusei*)	5 (1.6%)	0 (0.0%)	2 (10.5%)	0 (0.0%)	2 (3.39%)	9 (1.58%)
*Candida parapsilosis* complex	51 (15.8%)	13 (21. 7%)	4 (21.1%)	16 (14.7%)	23 (38.9%)	107 (18.8%)
*Candida lusitaniae*	5 (1.6%)	0 (0.0%)	0 (0.0%)	8 (7.34%)	2 (3.39%)	15 (2.63%)
*Candida tropicalis*	20 (6.2%)	1 (1.7%)	1 (5.3%)	4 (3.67%)	6 (10.12%)	32 (5.61%)
*Candida auris*	0 (0.0%)	0 (0.0%)	0 (0.0%)	0 (0.0%)	0 (0.0%)	0 (0.0%)
Other *Candida* species **	8 (2.5%)	1 (1.7%)	1 (5.3%)	0 (0.0%)	3 (5.1%)	13 (2.3%)
Total	323 (100.0%)	60 (100.0%)	19 (100.0%)	109 (100.0%)	59 (100.0%)	570 (100.0%)

* Data for the year 2017 were not available; ** *Candida guilliermondii*, *Candida kefyr*, *Candida lipolytica*, and *Candida slooffiae*.

**Table 2:** The percentage of susceptible *Candida* isolates (S and I categories, according to EUCAST criteria applicable for the respective year, intermediate isolates were included among susceptible strains) to antifungal drugs in candidemia cases across the studied hospitals (2017–2022).

Antifungal Drug	Hospital A	Hospital B	Hospital C	Hospital D	Hospital E	Total
*Candida albicans*						
AMB	100%	100%	100%	100%	90.9%	98.2%
FLC	98.63%	100%	85.7%	100%	90.9%	95.1%
VRC	98.63%	100%	83.3%	100%	90.9%	94.6%
ANI	99%	100%	- ^^	-	-	99.5%
*Nakaseomyces glabratus* (formerly *Candida glabrata*)						
AMB	100%	100%	100%	100%	90%	98%
FLC	93.2%	75%	-	93.1%	0	65.3%
VRC	IE	IE	IE	IE	IE	IE
ANI	100%	100%	- ^^	-	-	100%
*Pichia kudriavzevii* (formerly *Candida krusei*)						
AMB	100%	-	50%	100%	50%	75%
FLC	-	-	-	-	-	-
VRC	IE	-	IE	IE	IE	IE
ANI	100%	-	- ^^	-	-	100%
*Candida parapsilosis* complex						
AMB	100%	100%	100%	100%	100%	100%
FLC	87%	100%	100%	100%	69.6%	91.3%
VRC	92.2%	100%	100%	100%	60%	90.4%
ANI	86.3%	100%	- ^^	-	-	93.1%
*Candida tropicalis*						
AMB	100%	100%	100%	100%	100%	100%
FLC	100%	100%	0	100%	100%	80%
VRC	100%	-	0	100%	100%	75%
ANI	100%	-	- ^^	-	-	100%

AMB, amphotericin B; FLC, fluconazole; VRC, voriconazole; ANI, anidulafungin; IE—no EUCAST MIC interpretation criteria available; ^^ No tests were performed for anidulafungin, the values indicate susceptibility to caspofungin and micafungin, and no resistance to these drugs was detected.

**Table 3:** Antifungal susceptibility categories (S/I/R) and corresponding MIC ranges for *Candida* isolates by year (2017–2022) in Hospital A.

Antifungal Drug	*Candida* Species	Susceptibility Category *	Percentage of Isolates in Each Category (S/I/R) (MIC Ranges [mg/L])					
			2017	2018	2019	2020	2021	2022
FLC	Ca	S, I	93% (0.19–1)	100% (0.25–1)	100% (0.5–1)	100% (0.5–2)	100% (0.25–1)	100% (0.25–1)
		R	7% (16–256)	0	0	0	0	0
	Ng	S, I	92% (2–16)	100% (2–32)	94% (4–16)	100% (8–32)	86% (2–16)	90% (2–8)
		R	8% (256)	0	6% (256)	0	14% (32–256)	10% (128)
	Cpc	S, I	58% (0.5–2)	100% (0.5–1)	86% (0.5–2)	100% (0.5–2)	100% (0.5–2)	100% (0.5–1)
		R	42% (256)	0	14% (8)	0	0	0
VRC	Ca	S, I	93% (0.012–0.125)	100% (0.06–0.125)	100% (0.06)	100% (0.008–0.19)	100% (0.008–0.125)	100% (0.008–0.06)
		R	7% (1)	0	0	0	0	0
	Ng	IE	n/a (0.125–8)	n/a (0.125–0.25)	n/a (0.03–1)	n/a (0.125–0.25)	n/a (0.03–32)	n/a (0.03–1)
	Cpc	S, I	55% (0.125–0.25)	100% (0.016–0.125)	100% (0.094–0.125)	100% (0.125–0.25)	100% (0.008–0.125)	100% (0.008–0.016)
		R	45% (0.38–0.75)	0	0	0	0	0
AMB	Ca	S	100% (0.25–0.5)	100% (0.25–0.5)	100% (0.25–0.5)	100% (0.094–0.5)	100% (0.03–0.5)	100% (0.125–0.5)
		R	0	0	0	0	0	0
	Ng	S	100% (0.094–0.5)	100% (0.25–0.5)	100% (0.25–1)	100% (0.25–0.5)	100% (0.125–0.5)	100% (0.125–0.5)
		R	0	0	0	0	0	0
	Cpc	S	100% (0.016–0.5)	100% (0.25–0.5)	100% (0.03–0.5)	100% (0.25–1)	100% (0.125–0.5)	100% (0.125–0.5)
		R	0	0	0	0	0	0
ANI	Ca	S, I	100% (0.002–0.06)	100% (0.002–0.012)	94% (0.002–0.38)	100% (0.002–0.125)	100% (0.002–0.03)	100% (0.002–0.03)
		R	0	0	6% (1)	0	0	0
	Ng	S, I	100% (0.004–0.06)	100% (0.004–0.125)	100% (0.004–0.03)	100% (0.003–0.006)	100% (0.006–0.06)	100% (0.03–0.06)
		R	0	0	0	0	0	0
	Cpc	S, I	100% (0.002–2)	100% (0.38–1.5)	100% (0.25–2)	38% (1–2)	80% (0.19–4)	100% (0.125–1)
		R	0	0	0	62% (6–12)	20% (8)	0

MIC, minimum inhibitory concentration; S, susceptible; I, susceptible increased exposure; R, resistant; IE, insufficient evidence to categorize the isolate as S, I or R; AMB, amphotericin B; FLC, fluconazole; VRC, voriconazole; ANI, anidulafungin; Ca, *Candida albicans*; Ng, *Nakaseomyces glabratus*; Cpc, *Candida parapsilosis* complex; * MIC values were interpreted according to EUCAST guidelines valid in the respective years; n/a, not available.

## Data Availability

The data presented in this study are available on reasonable request from the first author. The data are not publicly accessible due to institutional privacy and data protection regulations.

## Supplementary Materials

The following supporting information can be downloaded at: https://www.mdpi.com/article/10.3390/jof12030212/s1. Table S1: Overview of microbiological diagnostic methods for candidemia used across hospital units; Table S2: Annual counts of Candida species isolated from candidemia cases in Hospital A (2017–2022).
