# Supplementary material for: Candidemia in Southern Poland (2017–2022): Multicenter Analysis of Species Distribution and Antifungal Susceptibility
Source: J Fungi (Basel). 2026 Mar 15;12(3):212. doi: 10.3390/jof12030212 (PMC13028304; doi:10.3390/jof12030212)
Supplement: Supplementary file 1 [file jof-12-00212-s001.zip › jof-4154536-supplementary.pdf]

Candidemia in Southern Poland (2017–2022): Multicenter Analysis of Species Distribution and Antifungal Susceptibility

Magdalena Namysł [1,†], Magdalena Skóra [2,†], Monika Pomorska-Wesołowska [3], Małgorzata Romanik [4], Wioletta Świątek-Kwapniewska [5,6], Piotr Serwacki [6], Iwona Pawłowska [7], Aldona Olechowska-Jarząb [1] and Jadwiga Wójkowska-Mach [2,5,*]

Table S1. Overview of microbiological diagnostic methods for candidemia used across hospital units.

**Methods**	**Hospital A**	**Hospital B**	**Hospital C**	**Hospital D**	**Hospital E**
Blood culture system	BACT/ALERT® 3D (bioMérieux, France), BACT/ALERT® VIRTUO® (bioMérieux, France)	BD BACTEC™ Mycosis (Becton, Dickinson)	BACT/ALERT® 3D (bioMérieux, France)	BD BACTEC™ Mycosis (Becton, Dickinson)	BACT/ALERT® 3D (bioMérieux, France)
Agar growth medium	Sabouraud glucose agare with chloramphenicol and gentamicin (Thermo Scientific™ Oxoid™, Germany) (bioMérieux, France)	Sabouraud glucose agare with chloramphenicol and gentamicin (Becton Dickinson) (bioMérieux, France)	Sabouraud glucose agare with chloramphenicol and gentamicin (GRASO, Biotech, Poland)	Sabouraud glucose agare with chloramphenicol and gentamicin (Becton Dickinson)	Sabouraud glucose agare with chloramphenicol and gentamicin (GRASO, Biotech, Poland), Chromagar Candida Plus (GRASO, Biotech, Poland)
Species identification method	VITEK® 2 YST (bioMérieux, France), VITEK® MS (bioMérieux, France)	BD Phoenix™ System M50 (Becton Dickinson)	VITEK® MS (bioMérieux, France)	MALDI Biotyper (Bruker Daltonics, Germany)	VITEK® MS (bioMérieux, France)
Antifungal drug susceptibility testing method	VITEK® 2 AST-YS08 (bioMérieux, France), Sensititre™ YeastOne™ YO10 (Thermo Fisher Scientific),	FUNGITEST® (Bio Rad, France), MICRONAUT AM	VITEK® 2 AST-YS08 (bioMérieux, France), MICRONAUT-AM (MERLIN Diagnostika,	ATB Fungus 3 INT (bioMérieux, France)	VITEK® 2 AST-YS08 (bioMérieux, France), Etest (bioMérieux)

	MICRONAUT-AM (MERLIN Diagnostika, Germany), Liofilchem® MIC Test Strip and RPMI agar (Liofilchem, Italy)	(MERLIN Diagnostika, Germany)	Germany), MICRONAUT-RPMI+MOPS+Glucose (MERLIN Diagnostika, Germany)		

Table S2. Annual counts of *Candida* species isolated from candidemia cases in Hospital A (2017–2022).

***Candida* species**	**Number of *Candida* strains isolated in individual years**						**Total**
	**2017**	**2018**	**2019**	**2020**	**2021**	**2022**	
Candida albicans	29	25	18	18	31	25	146
Candida dubliniensis	4	0	1	3	4	3	15
Nakaseomyces glabratus	13	14	17	5	14	10	73
Candida parapsilosis	12	7	7	8	8	6	48
Candida metapsilosis	0	0	0	0	2	0	2
Candida orthopsilosis	0	0	0	0	0	1	1
Candida tropicalis	3	2	3	2	6	4	20
Candida guilliermondii	0	0	1	0	1	1	3
Candida kefyr	0	0	1	1	1	0	3
Candida lipolytica	0	1	0	0	0	0	1
Candida lusitaniae	0	1	2	1	0	1	5
Candida slooffiae	0	0	0	0	0	1	1
Pichia kudriavzevii	2	0	1	0	1	1	5
**Total**	63	50	51	38	68	53	323
